# Novel therapeutic features of disulfiram against hepatocellular carcinoma cells with inhibitory effects on a disintegrin and metalloproteinase 10

**DOI:** 10.18632/oncotarget.24568

**Published:** 2018-04-10

**Authors:** Kaku Goto, Jun Arai, Anthony Stephanou, Naoya Kato

**Affiliations:** ^1^ The Advanced Clinical Research Center, The Institute of Medical Science, The University of Tokyo, Tokyo 108-8639, Japan; ^2^ Research Center for Hepatitis and Immunology, National Center for Global Health and Medicine, Chiba 272-8516, Japan; ^3^ Department of Medicine, Division of Gastroenterology, Showa University School of Medicine, Tokyo 142-8555, Japan; ^4^ Department of Gastroenterology and Nephrology, Chiba University, Graduate School of Medicine, Chiba 260-8670, Japan

**Keywords:** disulfiram, antabuse, HCC, ADAM10, MICA

## Abstract

Our previous genome-wide association study identified the anti-tumor ligand MHC class I polypeptide-related sequence A (*MICA*) as a susceptibility gene for hepatitis C virus-induced hepatocellular carcinoma (HCC). We subsequently proved that pharmacological restoration of membrane-bound MICA in HCC cells boosted natural killer cell-mediated anti-cancer effects, confirming that a MICA sheddase, a disintegrin and metalloproteinase 10 (ADAM10), is a therapeutic target. We here searched for approved drugs with inhibitory effects on ADAM10 *in vitro*, and the anti-alcoholism agent, disulfiram, was identified. Disulfiram elevated membrane-bound MICA levels and reduced production of soluble MICA, an immunological decoy, while simultaneously not having unfavorable off-target effects on natural killer cells and normal human hepatocytes. Functional analyses indicated a mode of non-zinc-binding inhibition of ADAM10 by disulfiram, which also suppressed HCC cell migration. These effects of disulfiram against HCC are expected to further the development of novel therapeutic regimens.

## INTRODUCTION

Hepatocellular carcinoma (HCC) is the third most common cause of cancer-related death, with incidence and mortality continuing to increase [1]. Although interventional methods for controlling the leading risk factors, hepatitis B virus (HBV) and hepatitis C virus (HCV), have improved, pharmacologically therapeutic and preventive options for HCC still remain limited [2]. Therefore, further development of safe and cost-effective regimens for management of HCC is an urgent need.

Our previous genome-wide association study (GWAS) identified the anti-tumor ligand MHC class I polypeptide-related sequence A (*MICA*) as a susceptibility gene for HCV-induced HCC [3]. Genetic risk of HCC was associated with MICA expression in chronic hepatitis C patients [4, 5] as well as patients with HBV infection [6], thereby indicating hypofunction of anti-HCC immunity by MICA insufficiency as a therapeutic target [7]. In line with this hypothesis, restoration of membrane-bound MICA (mMICA) levels in HCC cells augmented natural killer (NK) cell-mediated anti-cancer activity in our and other groups’ studies [2, 7], proving the concept of natural killer group 2D (NKG2D) signaling-mediated immunotherapy. We also demonstrated significant mMICA upregulation and reduction of levels of soluble MICA (sMICA), an immunological decoy, by an inhibitor of a disintegrin and metalloproteinase 10 (ADAM10) [2]. ADAM10 is reported to be a MICA sheddase [8] and a promoter of HCC cell proliferation, invasion, and migration, which is correlated with worse prognosis and shorter survival in patients [9]. Hence, we aimed to develop molecular pharmacological regulation of ADAM10 enzyme activity to modulate mMICA expression and have potential chemotherapeutic effects. We explored approved drugs, using a newly established *in vitro* assay system. Interestingly, a long-used anti-alcoholism drug, disulfiram (DSF), was identified and its cellular action was investigated.

## RESULTS

### DSF inhibits ADAM10 activity *in vitro*

**Figure 1:**
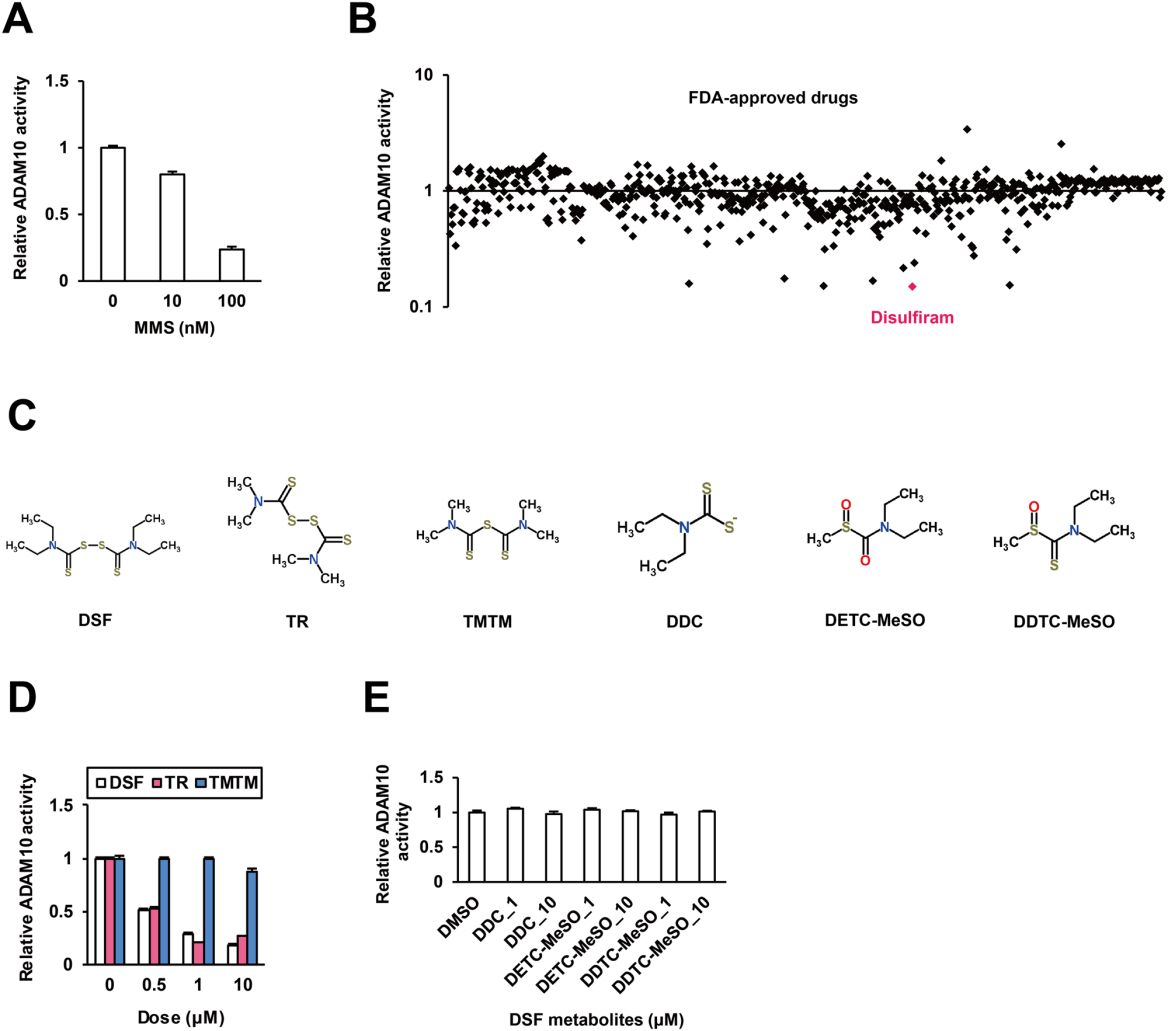
Inhibitory effects of DSF on ADAM10 *in vitro* **(A)** The *in vitro* ADAM10 enzyme assay system was validated using the known inhibitor MMS as a positive control. **(B)** The effects of 636 compounds in the FDA-Approved Drug Screen-well Library were tested *in vitro*. **(C)** The chemical structures of DSF and its analogues and metabolites, retrieved from the database ChemSpider. *In vitro* effects of **(D)** DSF, TR and TMTM at 0.5, 1, and 10 μM, and **(E)** DDC, DETC-MeSO and DDTC-MeSO at 1 and 10 μM.

To evaluate the enzymatic activity of ADAM10, we established a new *in vitro* assay system. Recombinant human ADAM10 (rhADAM10) protein was incubated with a fluorescent peptide substrate for fluorescence resonance energy transfer (FRET) analysis, as described in the Materials and Methods. RhADAM10-specific fluorescence was observed. Marimastat (MMS), an ADAM10 inhibitor [10], suppressed fluorescence in a dose-dependent manner (Figure 1A), validating the reporter system. We subsequently tested 636 FDA-approved drugs *in vitro*; six drugs demonstrated inhibitory effects of more than 80% in the primary screen, and an anti-alcoholism drug, DSF, was the top hit (Figure 1B).

Then we investigated the inhibitory action of DSF (Figure 1C) in detail. DSF moderately suppressed ADAM10 activity in a dose-dependent manner at dosages below clinical concentrations (Figure 1D), replicating the results of the primary screen. Next, to identify the functional groups of DSF responsible for the inhibitory activity, two close analogues of DSF, tetramethylthiuram disulfide (TR) and tetramethylthiuram monosulfide (TMTM), were investigated (Figure 1C). TR exhibited inhibitory effects but TMTM did not (Figure 1D), indicating the importance of the disulfide bond. DSF metabolites diethyldithiocarbamate (DDC), S-methyl-N,N-diethylthiocarbamoyl sulfoxide (DETC-MeSO), and S-methyl-N,N-diethyldithiocarbamate-sulfoxide (DDTC-MeSO) (Figure 1C) were also tested, and at the effective doses of DSF and TR, no inhibition was observed (Figure 1E). Thus, the disulfide bond-based conformational integrity is suggested to be important for the inhibition of ADAM10 activity by DSF.

### DSF suppresses sMICA production and selectively upregulates mMICA

**Figure 2:**
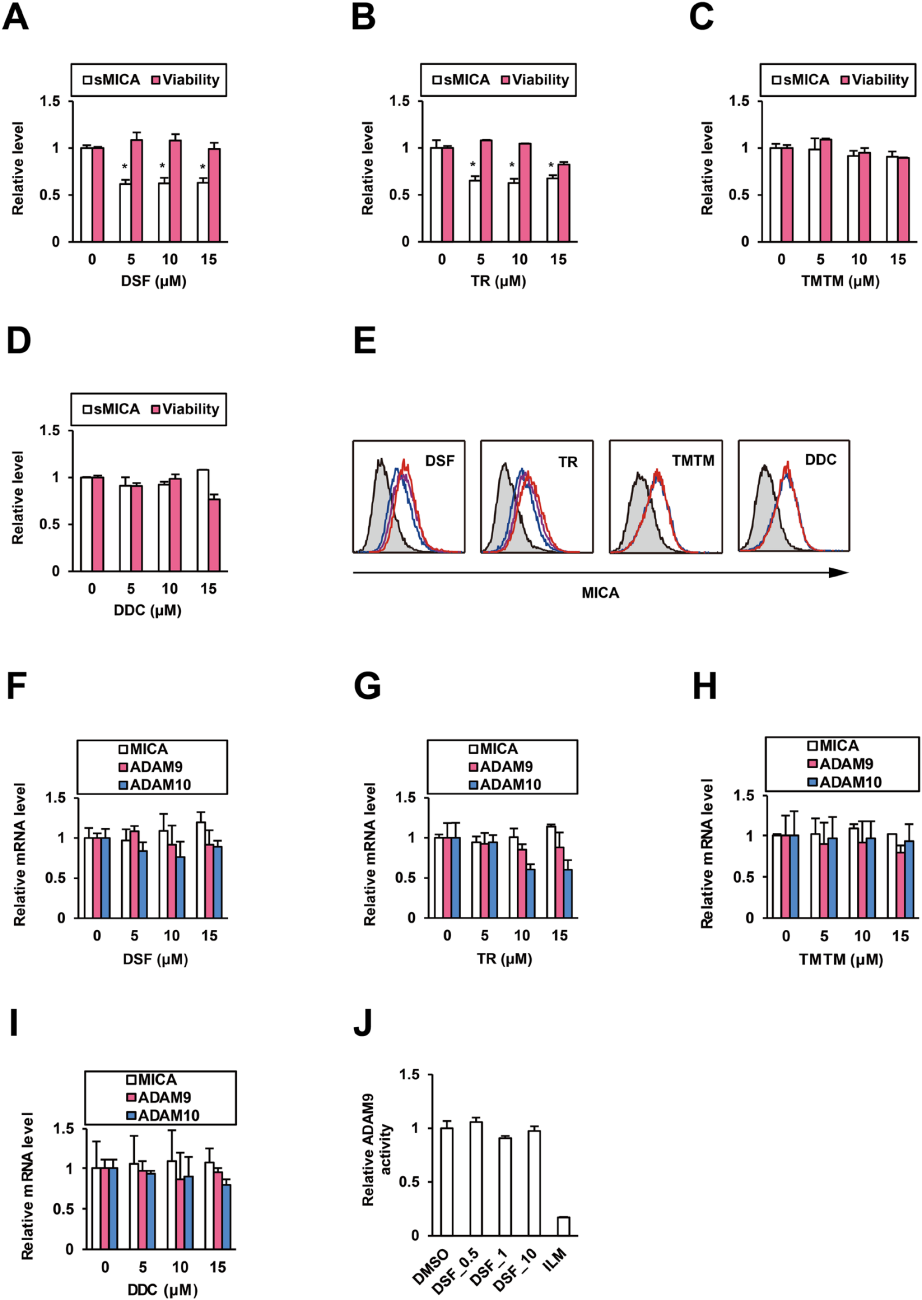
DSF reduces sMICA production and enhances mMICA levels After the treatment of PLC/PRF/5 cells for 48 h, the effects of **(A)** DSF, **(B)** TR, **(C)** TMTM, and **(D)** DDC on sMICA production and cell viability were examined. ^*^P < 0.05 by the Welch t-test. **(E)** After the treatment of PLC/PRF/5 cells for 72 h, the effects of DSF (0, 5, and 15 μM in blue, purple, and red, respectively), TR (0, 5, and 15 μM in blue, purple, and red, respectively), TMTM (0 and 15 μM in blue and red, respectively), and DDC (0 and 15 μM in blue and red, respectively) on mMICA levels were evaluated flow cytometrically, with the isotype controls displayed as gray histograms. After the treatment of PLC/PRF/5 cells for 48 h, the effects of **(F)** DSF, **(G)** TR, **(H)** TMTM, and **(I)** DDC on relative mRNA levels of *MICA*, *ADAM9*, and *ADAM10* were determined by qRT-PCR with normalization to *GAPDH*. **(J)** Effects of DSF at 0.5, 1, and 10 μM and ILM at 1 μM on ADAM9 were tested *in vitro*.

We examined the effects of DSF on MICA expression in HCC cells. PLC/PRF/5 cells were treated with DSF or the analogues for 48 h, and then cell viability was tested and culture supernatants were collected for ELISA. At noncytotoxic doses, DSF (Figure 2A) and TR (Figure 2B) suppressed the relative sMICA level but TMTM (Figure 2C) and DDC (Figure 2D) did not, and the inhibitory effect of DSF was abrogated (Supplementary Figure 1A) by the knockdown of ADAM10 (Supplementary Figure 1B). Conversely, mMICA levels in PLC/PRF/5 cells treated for 72 h were increased by DSF and TR in a dose-dependent manner, but not by TMTM and DDC (Figure 2E and Supplementary Table 1); the stimulatory effects of DSF on mMICA were also observed in additional hepatoma cell lines, Li7 and HLE (Supplementary Figure 1C). Concomitantly, NK cell-mediated cytotoxicity to DSF-treated PLC/PRF/5 cells was potentiated in coculture (Supplementary Figure 1D). Therefore, DSF was suggested to enhance mMICA levels and suppress sMICA production with enzymatic inhibition of ADAM10, leading to anti-cancer activity of NK cells. To confirm that ADAM10 activity is specifically targeted by DSF, we evaluated the effects of DSF on transcription of *MICA* and *ADAM10*. DSF (Figure 2F) and TR (Figure 2G), as well as TMTM and DDC (Figure 2H and 2I), did not significantly affect *MICA* mRNA levels. TR alone exhibited a moderate suppression of *ADAM10* mRNA levels (Figure 2G) while DSF (Figure 2F) and the other compounds (Figure 2H and 2I) had no effect.

ADAM9 has also been reported to be a MICA sheddase in HCC cells [11], and suppression of ADAM9 by DSF was investigated simultaneously. Similar to that for ADAM10, an *in vitro* ADAM9 FRET assay system was developed, as described in the Materials and Methods; an ADAM9 inhibitor, ilomastat (ILM) [12], suppressed ADAM9 activity while DSF did not (Figure 2J). *ADAM9* mRNA levels were not altered by DSF (Figure 2F), TR (Figure 2G), TMTM, or DDC (Figure 2H and 2I). These results indicate that ADAM9 is not targeted either transcriptionally or enzymatically by DSF.

**Figure 3:**
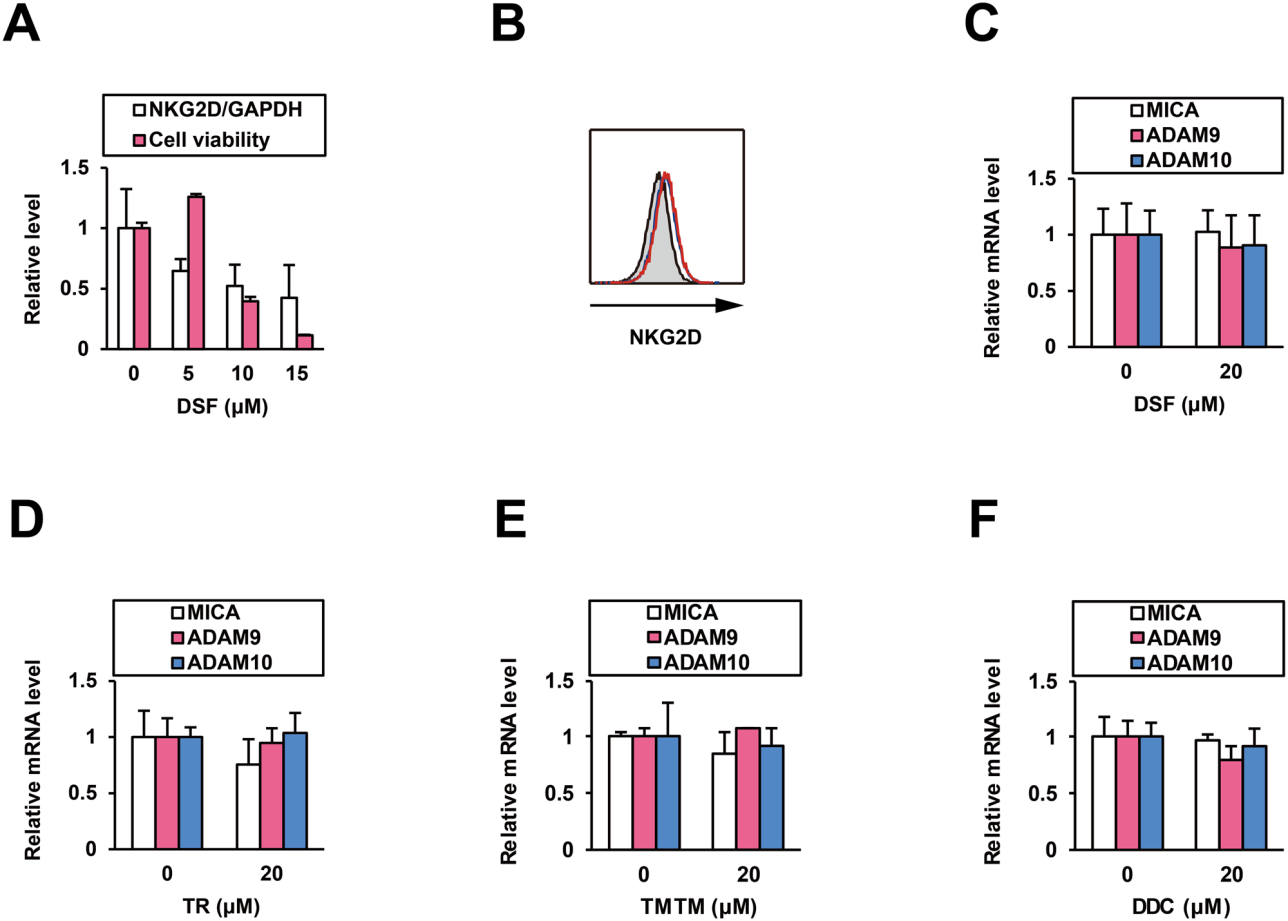
Effects of DSF on NK cells and normal human hepatocytes After the treatment of NK92MI cells for 48 h, **(A)** the effects of DSF at 5, 10, and 15 μM on relative *NKG2D* mRNA levels were examined by qRT-PCR with normalization to *GAPDH*, and **(B)** the effects of DSF (0 and 5 μM in blue and red, respectively, with the isotype control displayed as a gray histogram) on cell surface protein expression of NKG2D was examined by flow cytometry; simultaneously, (A) the effects of DSF at the indicated doses on cell viability was monitored. After the treatment of PXB cells for 48 h, effects of **(C)** DSF, **(D)** TR, **(E)** TMTM, and **(F)** DDC were examined by qRT-PCR with normalization to *GAPDH*.

To investigate potentially unfavorable immunological effects of DSF, we tested its influence on NK cells. In cells from a NK cell line (NK92MI) treated with DSF for 48 h, *NKG2D* mRNA levels were intermediately decreased (Figure 3A) but its cell surface protein level was not altered (Figure 3B). Cell viability was significantly reduced at higher doses (Figure 3A). At 5 μM, an effective dose for ADAM10 inhibition, DSF was indicated to function without disturbance of the NKG2D signaling and NK cells.

We also examined the impact of DSF on normal human hepatocytes. In PXB cells from chimeric mice with a humanized liver [13] treated for 48 h, neither DSF (Figure 3C), TR (Figure 3D), TMTM, nor DDC (Figure 3E and 3F), at the noncytotoxic dose [14], altered the mRNA levels of *MICA*, *ADAM9*, or *ADAM10*. This confirms that DSF is free from unwanted induction of the immunoactivating ligand on normal hepatocytes, which would specifically increase mMICA levels with ADAM10 enzyme inhibition.

### Mechanism of ADAM10 inhibition by DSF in a combination treatment

**Figure 4:**
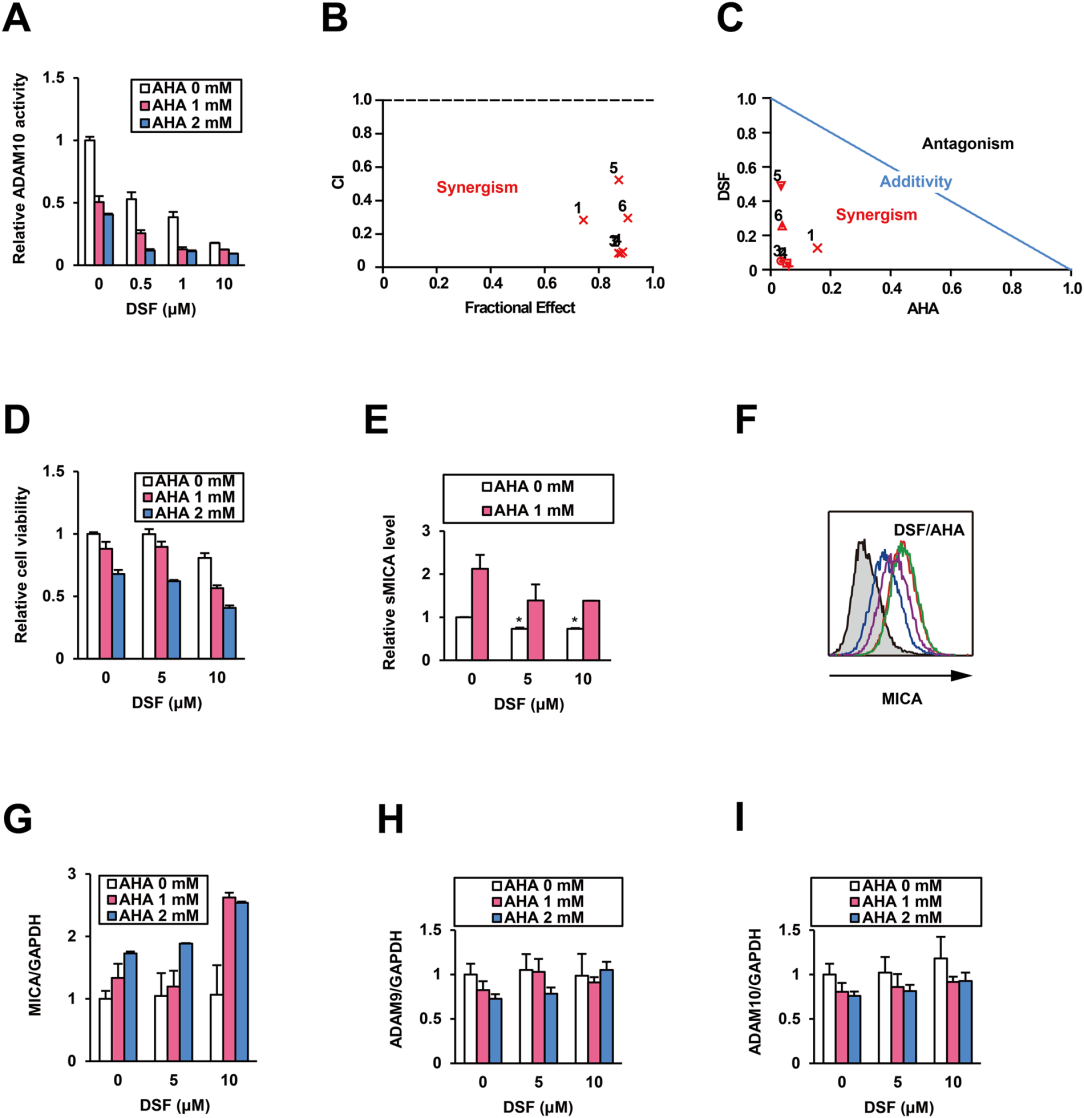
Combination treatment with DSF and AHA Effects of the combination treatment with DSF and AHA on ADAM10 **(A)** were tested *in vitro*, and synergism was evaluated by **(B)** CI and **(C)** normalized isobologram at the following ratios: 1) 0.5:1, 2) 0.5:2, 3) 1:1, 4) 1:2, 5) 10:1, and 6) 10:2 (DSF (μM):AHA (mM)). After the treatment of PLC/PRF/5 cells with DSF and AHA at the indicated doses for 48 h, **(D)** relative cell viability and **(E)** sMICA levels were measured. ^*^P < 0.05 by the Welch t-test. **(F)** The mMICA levels in PLC/PRF/5 cells were examined by flow cytometry after no treatment (Blue), monotreatment with DSF (purple) or AHA (red), and cotreatment with DSF and AHA (green) for 72 h, with the isotype control displayed as a gray histogram. After cotreatment with DSF and AHA at the indicated doses for 48 h, relative mRNA levels of *MICA* **(G)**, *ADAM9* **(H)**, and *ADAM10* **(I)** were measured by qRT-PCR with normalization to *GAPDH*.

To investigate the mechanism of ADAM10 inhibition by DSF, we performed an *in vitro* combination assay with a well-known zinc-binding probe, acetohydroxamic acid (AHA) [10]. The dose-dependent inhibition of ADAM10 activity by DSF was further reinforced in the presence of AHA (Figure 4A). All calculated combination indices (CIs) were less than 1.0 (Figure 4B), indicating synergistic effects [10] of combining DSF and AHA. This result was also supported by the analysis of normalized isobologram (Figure 4C). The data at least indicates a mode of non-zinc-binding inhibition of ADAM10 by DSF, as DSF and AHA did not compete with each other [10].

On the basis of the moderate inhibition of ADAM10 by AHA (Figure 4A), in addition, we investigated the effects of AHA on HCC cells by methods similar to those used to evaluate DSF. As expected, AHA modestly decreased viabilities of PLC/PRF/5 cells after a 48-h treatment, which was more pronounced when applied in combination with DSF (Figure 4D). Interestingly, AHA elevated sMICA levels in PLC/PRF/5 cells after a 48-h treatment, in contrast to the downregulation induced by DSF (Figure 4E). This indicates that AHA is an inducer of MICA expression. Flow cytometric analysis of the cells treated with AHA for 72 h predictably demonstrated the enhancement of mMICA levels, and this was slightly reinforced by DSF (Figure 4F). The same pattern was found for *MICA* mRNA levels as well (Figure 4G), but the expression of *ADAM9* (Figure 4H) and *ADAM10* (Figure 4I) was not affected by AHA, with or without DSF. Thus, the potential utility of AHA, an approved urease inhibitor Lithostat, for mMICA upregulation and HCC cell suppression was also found during this combination assay.

### DSF possesses anti-migratory activity

**Figure 5:**
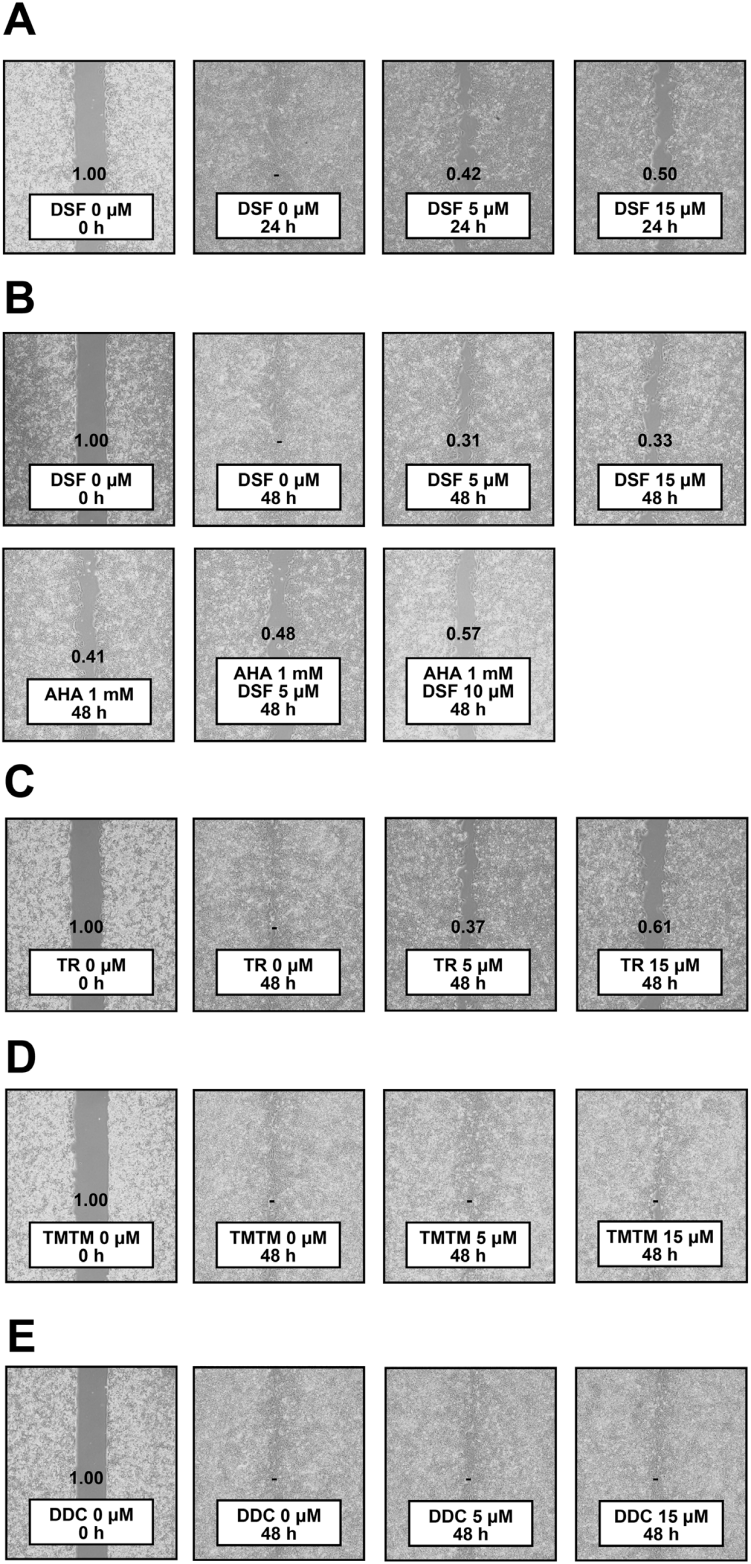
Wound closure assay **(A)** PLC/PRF/5 cells were treated with DSF for 24 h. PLC/PRF/5 cells were pretreated with mitomycin at 10 μg/mL for 1 h, followed by treatment with DSF and/or **(B)** AHA, **(C)** TR, **(D)** TMTM and **(E)** DDC at the indicated doses for 48 h. Relative wound sizes to the controls are indicated individually; dashes denote no detection of wounds.

Since ADAM10 is notorious for the ability to promote migration of HCC cells [9, 15], we also tested anti-migratory effects of DSF. Wound closure assays demonstrated that DSF exerted inhibitory effects on PLC/PRF/5 cell proliferation and migration (Figure 5A). The assays were subsequently performed after pretreatment with mitomycin, and the anti-migratory effects of DSF were still observed, slightly increased by AHA, a reported migration inhibitor (Figure 5B); the inhibitory effects of DSF were observed in additional hepatoma cell lines, Li7 and HLE, as well (Supplementary Figure 2). TR also showed effects (Figure 5C) while TMTM (Figure 5D) and DDC (Figure 5E) did not. DSF was therefore suggested to possess anti-migratory activity in relation to ADAM10 inhibition, as the effects were associated with *in vitro* inhibitory activity.

## DISCUSSION

**Figure 6:**
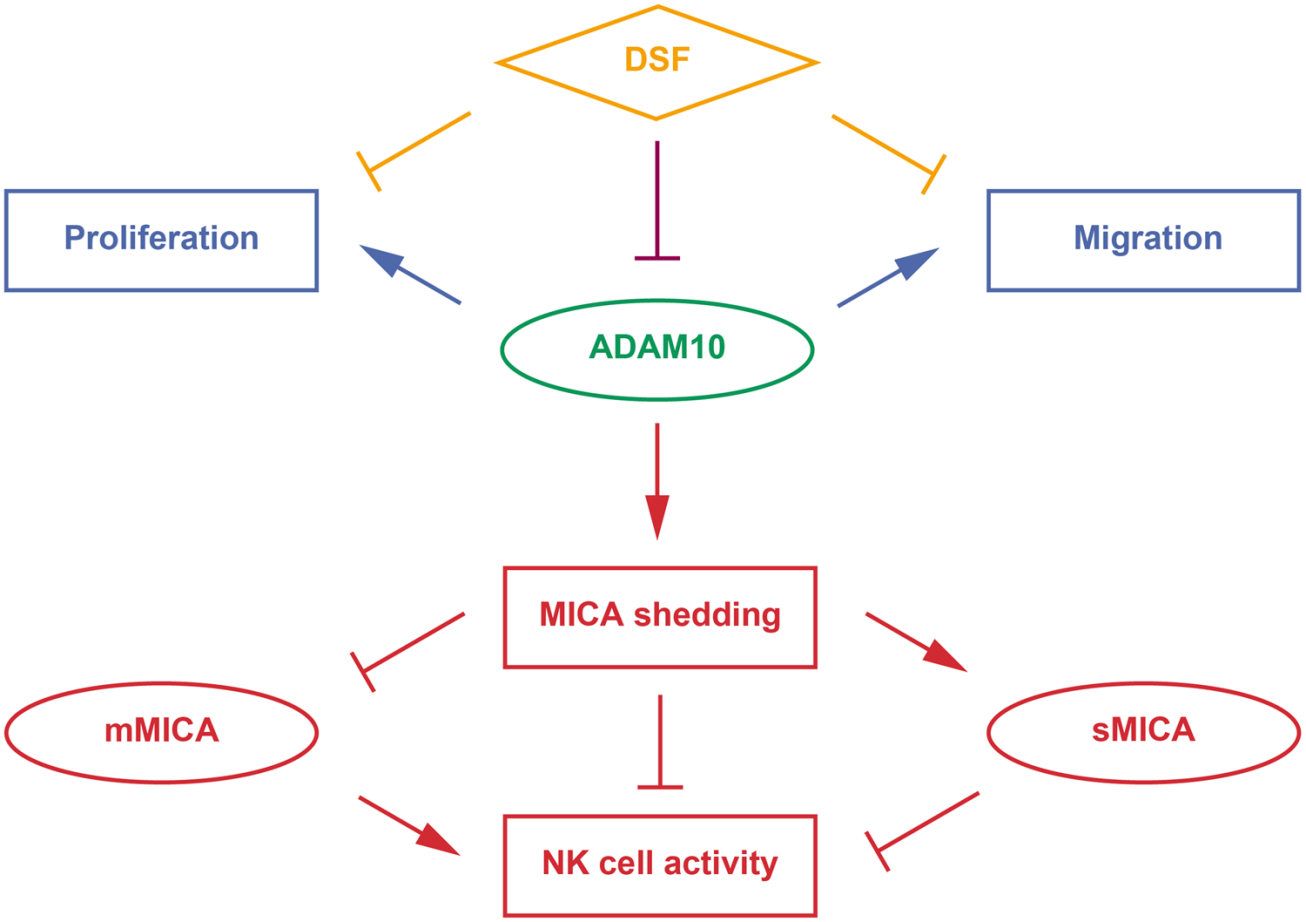
Therapeutic modes of DSF targeting ADAM10 DSF has been increasingly recognized to possess anti-cancer effects through various pathways (orange). Here, DSF was discovered to exert inhibitory effects on the enzymatic activity of ADAM10 (purple), which promotes MICA shedding, enhances sMICA production and decreases mMICA level, and suppresses anti-HCC activity of NK cells (red, immunotherapeutic targets). ADAM10 also promotes HCC cell proliferation and migration (blue, chemotherapeutic targets).

Our preclinical proof-of-concept study [2] based on the previous GWAS [3] suggested the anti-HCC immunotherapeutic validity of targeting ADAM10 to increase mMICA expression. Besides the discovery and observation of inhibitory effects of DSF on ADAM10 activity, pleiotropy of the enzyme was reflected in the various chemotherapeutic properties of DSF. Novel and multiple effective chemoimmunotherapeutic strategies can be considered based on the unique properties of DSF and ADAM10 (Figure 6).

Following reports in several cancer cell lines [16], ADAM10 was also found to be overexpressed and to shed mMICA in HCC cells [8]. Pharmacological modulation of ADAM10 has proven to be effective. Both transcriptional downregulation [8] and enzymatic inhibition of ADAM10 [2] enhanced mMICA levels and reduced sMICA production in HCC cells, leading to the augmentation of NK cell-mediated cytotoxicity. Additionally, DSF did not upregulate *MICA* expression in normal human hepatocytes, with very low protein levels of MICA and ADAM10 in contrast to HCC cells [8, 17, 18], or interfere with *NKG2D* expression in NK cells (Figure 3A and 3B), which indicated selective enhancement of mMICA levels in HCC cells and efficient transduction of signaling for NK cell activation. Therefore, DSF is expected to possess immunotherapeutic properties without unfavorable off-target effects.

Mechanistically, possible non-zinc-binding inhibition was indicated from analysis of ADAM10 inhibition by DSF, implying that coadministration of DSF with chelating drugs could be an attractive strategy. Furthermore, AHA significantly elevated MICA expression levels; through zinc-binding properties, hydroxamic acids are known to inhibit enzymatic activities of zinc-metalloproteins such as Matrix Metallo Proteinase (MMP) and histone deacetylases (HDACs) [19]. In our recent study, HDAC inhibitor (HDACi) suberoylanilide hydroxamic acid, also known as vorinostat (VOR), strongly enhanced MICA expression, potentiating NK cell-mediated anti-HCC activity in cell culture and *in vivo* [2]. Here, the pronounced induction of mMICA levels by AHA, supposedly due to ADAM10 inhibition and whether or not HDACs are inhibited as well, is of practical use and of interest for further mechanistic investigation. Accordingly, development and utilization of hydroxamic acids dually targeting ADAM10 and HDACs, as implied by molecules against MMPs and HDACs [20], is warranted. Thus, therapeutic methods using DSF, DSF analogues/derivatives, or better potent ADAM10 inhibitors and hydroxamic acids could achieve potent immunotherapeutic effects by robust elevation of mMICA levels and *MICA* transcription, as well as selective inhibition of MICA shedding; importance of strategic surveillance and management of sMICA was learned as well from a series of clinicogenetic studies [3, 5, 6].

Meanwhile, ADAM10 has been recognized to promote cell migration in multiple cancers [21] including HCC [9, 15], and so inhibition of ADAM10 has been demonstrated to have anti-migratory effects. In this study, HCC cell migration was suppressed by DSF and TR, as well as AHA, but not by TMTM and DDC (Figure 5), supporting the validity of ADAM10 enzyme inhibition protecting against HCC progression. Considering that DSF suppresses migratory properties of various cancer types through targets other than ADAM10 [22], it can be an even more attractive agent.

ADAM10 is also known to promote cell growth in HCC [9, 15] and other cancers [21]. We recently showed inhibitory effects of DSF and TR on HCC cell proliferation [14], and in the current study, AHA exerted anti-HCC effects as well (Figure 4D). This strengthens the concept of inhibiting ADAM10 in order to suppress HCC. DSF is also capable of exerting effects targeting many diverse molecules [23], as represented by apoptosis induction through the reactive oxygen species-p38 pathway [24]. Our analyses demonstrated that DDC, a DSF metabolite shown to lack inhibitory effects on ADAM10 enzyme activity (Figure 1E), also decreased HCC cell viability [14], suggesting a further potential effect of DSF against HCC.

Thanks to such chemotherapeutic features against cancer cell proliferation and migration as discussed above and recently proved even in a nationwide epidemiological study of Danish patients with cancer [25], the anti-cancer properties of DSF are becoming increasingly appreciated (Supplementary Table 2) and tested in clinical trials across cancer types [23]. Our data describing ADAM10 inhibition has uncovered another aspect of DSF as an anti-cancer agent. At relatively lower doses, DSF suppressed MICA shedding (Figure 2), and cytotoxicity was not observed until higher doses were applied [14], indicating the drug action gradually switched from immunotherapeutic to chemotherapeutic modes. The therapeutic activities of DSF were HCC-cell selective, as was seen in the inhibition of MICA shedding (Figure 2 and 3) and cell growth [14], and this dual action is thought to be safely effectuated. Besides, the induction of MICA expression and inhibition of HCC cell viability by AHA were of particular interest as the compound is an approved urease inhibitor Lithostat. The enhanced effects of DSF in the presence of AHA suggest chemotherapeutic potential of a DSF-based regimen with the addition of approved drugs capable of inducing mMICA expression and inhibiting HCC cell proliferation, such as VOR [2]. This is expected to accelerate implementation of practical treatment options using available clinical agents.

Following a breakthrough in cancer immunotherapy [26] and continuous development of new molecularly targeted therapies [27], combined therapies are drawing more attention [28]. Recently, significant and synergistic suppression of tumor growth by the combination of anti-programmed cell death protein 1 antibodies and an FDA-approved receptor tyrosine kinase inhibitor, sunitinib, in a murine model of human HCC was reported [29]. The SHELTER study also exhibited a high disease control rate in HCC patients treated by an HDACi, resminostat, and sorafenib [30], capable of inducing *MICA* expression [2] and suppressing ADAM9-mediated mMICA shedding [11], respectively, suggesting chemoimmunotherapeutic effects [31]. Similarly, DSF, which has been indicated to possess both chemotherapeutic and immunotherapeutic properties, and/or its analogues may be used as treatments. The data in this study would lead to the development of new, safe, and cost-effective anti-HCC regimens.

## MATERIALS AND METHODS

### Compounds and cells

DSF was purchased from Selleckchem (Houston, TX, USA). TR, TMTM, Sodium diethyldithiocarbamate trihydrate, and MMS were purchased from Sigma-Aldrich (St. Louis, MO, USA). AHA and mitomycin were purchased from Wako Pure Chemical Industries (Osaka, Japan). ILM was purchased from Focus Biomolecules (Plymouth Meeting, PA, USA). DETC-MeSO and DDTC-MeSO were purchased from Santa Cruz Biotechnology (Santa Cruz, CA, USA). The FDA-Approved Drug Screen-well Library and Cell Counting Kit (CCK)-8 were obtained from Enzo Life Sciences (Farmingdale, NY, USA) and Dojindo (Kumamoto, Japan), respectively. PLC/PRF/5 cells were authenticated by the short tandem repeat method (Bex, Tokyo, Japan) in 2016 and cultured in Dulbecco’s modified Eagle medium containing fetal bovine serum according to the protocol (www.atcc.org/Products/All/CRL-8024.aspx#culturemethod) of American Type Culture Collection (ATCC) (Manassas, VA, USA). NK92MI cells were also obtained from ATCC and cultured in Alpha Minimum Essential medium containing horse and fetal bovine serums following the protocol (www.atcc.org/Products/All/CRL-2408.aspx#culturemethod). PXB cells were purchased from Phoenix Bio (Hiroshima, Japan). Cells were cultured at 37°C and 5 % CO_2_.

### *In vitro* ADAM assay

RhADAM9 (R&D systems, Minneapolis, MN) was incubated with a fluorescent peptide substrate (BioZyme, Apex, NC, USA), at 20 μg/mL and 10 μM, respectively, in the presence of DMSO or a compound according to the manufacturer’s instructions. After reacting for 24 h at 37°C, fluorescent signals were measured, and relative enzymatic activities were calculated. RhADAM10 (R&D systems) was incubated with a fluorescent peptide substrate (BioZyme), at 0.5 μg/mL and 10 μM, respectively, in the presence of DMSO or a compound according to the manufacturer’s instructions. After reacting for 2 h at 37°C, fluorescent signals were measured, and relative enzymatic activities were calculated.

### Analysis of drug combinations

Synergism between DSF and AHA was calculated using CalcuSyn software (Biosoft, Ferguson, MO, USA) based on the method of Chou and Talalay [32] as described previously [33].

### Quantitative reverse transcription-polymerase chain reaction

Relative mRNA levels were quantified as previously described [34], using the following primer sets: MICA-F 5'-CTTCCTGCTTCTGGCTGGCATC-3' and MICA-R 5'- CAGGGTCATCCTGAGGTCCTTTC-3' for *MICA*, ADAM9-F 5'-TCCATTGCTCTTAGCGACTGT-3' and ADAM9-R 5'-GGGGTTCAATCCCATAACTCG-3' for *ADAM9*, ADAM10-F 5'-ATGGGAGGTCAGTATGGGAATC-3' and ADAM10-R 5'-ACTGCTCTTTTGGCACGCT-3' for *ADAM10*, NKG2D-F 5'-GAGTGATTTTTCAACACGATGGC-3' and NKG2D-R 5'-ACAGTAACTTTCGGTCAAGGGAA-3' for *NKG2D*, and GAPDH-F2 5'-AAGGTGAAGGTCGGAGTCAAC-3' and GAPDH-R2 5'-GGGGTCATTGATGGCAACAATA-3' for glyceraldehyde-3-phosphate dehydrogenase (*GAPDH*), with the values of *MICA*, *ADAM9*, *ADAM10*, and *NKG2D* normalized to that of *GAPDH*.

### Flow cytometry

Flow cytometry was performed as described previously [2], using the following antibodies (R&D Systems) according to the manufacturer’s protocol: Alexa 488-conjugated mouse IgG isotype control, human MICA, or human NKG2D antibody.

### ELISA

ELISA was performed as described previously [2] using MICA ELISA Kit (Diaclone, Besançon, France) according to the manufacturer’s protocol.

### Wound closure assay

Using Culture-Insert (Ibidi, Martinsried, Germany), the procedure was performed according to the manufacturer’s protocol. Briefly, 70 μL of PLC/PRF/5 cell suspension at 8 × 10^5^ cells/mL was applied into each well, and 24 h later the Culture-Insert was removed. After the pretreatment with mitomycin at 10 μg/mL for 1 h, cells were incubated in the presence of compounds for 48 h. Wound areas were measured by Image J (NIH, Bethesda, MD, USA).

### Statistical analysis

The bar graphs of the *in vitro* ADAM10 assay, ELISA, cell viability assay, and qRT-PCR are presented as means ± SD. A Welch t-test was performed for statistical analysis of ELISA results.

## SUPPLEMENTARY MATERIALS FIGURES AND TABLES



## Abbreviations

DSF: Disulfiram
TR: tetramethylthiuram disulfide
TMTM: tetramethylthiuram monosulfide
DDC: diethyldithiocarbamate
DETC-MeSO: S-methyl-N,N-diethylthiocarbamoyl sulfoxide
DDTC-MeSO: S-methyl-N,N-diethyldithiocarbamate-sulfoxide
MICA: MHC class I polypeptide-related sequence A

## References

[1] Singh S, Singh PP, Roberts LR, Sanchez W (2014). Chemopreventive strategies in hepatocellular carcinoma. Nat Rev Gastroenterol Hepatol.

[2] Goto K, Annan DA, Morita T, Li W, Muroyama R, Matsubara Y, Ito S, Nakagawa R, Tanoue Y, Jinushi M, Kato N (2016). Novel chemoimmunotherapeutic strategy for hepatocellular carcinoma based on a genome-wide association study. Sci Rep.

[3] Kumar V, Kato N, Urabe Y, Takahashi A, Muroyama R, Hosono N, Otsuka M, Tateishi R, Omata M, Nakagawa H, Koike K, Kamatani N, Kubo M (2011). Genome-wide association study identifies a susceptibility locus for HCV-induced hepatocellular carcinoma. Nat Genet.

[4] Huang CF, Huang CY, Yeh ML, Wang SC, Chen KY, Ko YM, Lin CC, Tsai YS, Tsai PC, Lin ZY, Chen SC, Dai CY, Huang JF (2017). Genetics Variants and Serum Levels of MHC Class I Chain-related A in Predicting Hepatocellular Carcinoma Development in Chronic Hepatitis C Patients Post Antiviral Treatment. EBioMedicine.

[5] Lo PH, Urabe Y, Kumar V, Tanikawa C, Koike K, Kato N, Miki D, Chayama K, Kubo M, Nakamura Y, Matsuda K (2013). Identification of a functional variant in the MICA promoter which regulates MICA expression and increases HCV-related hepatocellular carcinoma risk. PLoS One.

[6] Kumar V, Yi Lo PH, Sawai H, Kato N, Takahashi A, Deng Z, Urabe Y, Mbarek H, Tokunaga K, Tanaka Y, Sugiyama M, Mizokami M, Muroyama R (2012). Soluble MICA and a MICA variation as possible prognostic biomarkers for HBV-induced hepatocellular carcinoma. PLoS One.

[7] Goto K, Kato N (2015). MICA SNPs and the NKG2D system in virus-induced HCC. J Gastroenterol.

[8] Kohga K, Takehara T, Tatsumi T, Miyagi T, Ishida H, Ohkawa K, Kanto T, Hiramatsu N, Hayashi N (2009). Anticancer chemotherapy inhibits MHC class I-related chain a ectodomain shedding by downregulating ADAM10 expression in hepatocellular carcinoma. Cancer Res.

[9] Yuan S, Lei S, Wu S (2013). ADAM10 is overexpressed in human hepatocellular carcinoma and contributes to the proliferation, invasion and migration of HepG2 cells. Oncol Rep.

[10] Madoux F, Dreymuller D, Pettiloud JP, Santos R, Becker-Pauly C, Ludwig A, Fields GB, Bannister T, Spicer TP, Cudic M, Scampavia LD, Minond D (2016). Discovery of an enzyme and substrate selective inhibitor of ADAM10 using an exosite-binding glycosylated substrate. Sci Rep.

[11] Kohga K, Takehara T, Tatsumi T, Ishida H, Miyagi T, Hosui A, Hayashi N (2010). Sorafenib inhibits the shedding of major histocompatibility complex class I-related chain A on hepatocellular carcinoma cells by down-regulating a disintegrin and metalloproteinase 9. Hepatology.

[12] Kwan JC, Eksioglu EA, Liu C, Paul VJ, Luesch H (2009). Grassystatins A-C from marine cyanobacteria, potent cathepsin E inhibitors that reduce antigen presentation. J Med Chem.

[13] Kakuni M, Yamasaki C, Tachibana A, Yoshizane Y, Ishida Y, Tateno C (2013). Chimeric mice with humanized livers: a unique tool for *in vivo* and *in vitro* enzyme induction studies. Int J Mol Sci.

[14] Goto K, Kato N, Chung RT (2016). Anti-hepatocellular carcinoma properties of the anti-alcoholism drug disulfiram discovered to enzymatically inhibit the AMPK-related kinase SNARK *in vitro*. Oncotarget.

[15] Liu S, Zhang W, Liu K, Ji B, Wang G (2015). Silencing ADAM10 inhibits the *in vitro* and *in vivo* growth of hepatocellular carcinoma cancer cells. Mol Med Rep.

[16] Waldhauer I, Goehlsdorf D, Gieseke F, Weinschenk T, Wittenbrink M, Ludwig A, Stevanovic S, Rammensee HG, Steinle A (2008). Tumor-associated MICA is shed by ADAM proteases. Cancer Res.

[17] Kohga K, Takehara T, Tatsumi T, Ohkawa K, Miyagi T, Hiramatsu N, Kanto T, Kasugai T, Katayama K, Kato M, Hayashi N (2008). Serum levels of soluble major histocompatibility complex (MHC) class I-related chain A in patients with chronic liver diseases and changes during transcatheter arterial embolization for hepatocellular carcinoma. Cancer Sci.

[18] Liu S, Liu K, Zhang W, Wang Y, Jin Z, Jia B, Liu Y (2016). miR-449a inhibits proliferation and invasion by regulating ADAM10 in hepatocellular carcinoma. Am J Transl Res.

[19] Roche J, Bertrand P (2016). Inside HDACs with more selective HDAC inhibitors. Eur J Med Chem.

[20] Pal D, Saha S (2012). Hydroxamic acid - A novel molecule for anticancer therapy. J Adv Pharm Technol Res.

[21] Mochizuki S, Okada Y (2007). ADAMs in cancer cell proliferation and progression. Cancer Sci.

[22] Jiao Y, Hannafon BN, Ding WQ (2016). Disulfiram's Anticancer Activity: Evidence and Mechanisms. Anticancer Agents Med Chem.

[23] Triscott J, Rose Pambid M, Dunn SE (2015). Concise review: bullseye: targeting cancer stem cells to improve the treatment of gliomas by repurposing disulfiram. Stem Cells.

[24] Chiba T, Suzuki E, Yuki K, Zen Y, Oshima M, Miyagi S, Saraya A, Koide S, Motoyama T, Ogasawara S, Ooka Y, Tawada A, Nakatsura T (2014). Disulfiram eradicates tumor-initiating hepatocellular carcinoma cells in ROS-p38 MAPK pathway-dependent and -independent manners. PLoS One.

[25] Skrott Z, Mistrik M, Andersen KK, Friis S, Majera D, Gursky J, Ozdian T, Bartkova J, Turi Z, Moudry P, Kraus M, Michalova M, Vaclavkova J (2017). Alcohol-abuse drug disulfiram targets cancer via p97 segregase adaptor NPL4. Nature.

[26] Couzin-Frankel J (2013). Breakthrough of the year 2013. Cancer immunotherapy. Science.

[27] Brown C (2016). Targeted therapy: An elusive cancer target. Nature.

[28] Gotwals P, Cameron S, Cipolletta D, Cremasco V, Crystal A, Hewes B, Mueller B, Quaratino S, Sabatos-Peyton C, Petruzzelli L, Engelman JA, Dranoff G (2017). Prospects for combining targeted and conventional cancer therapy with immunotherapy. Nat Rev Cancer.

[29] Li G, Liu D, Cooper TK, Kimchi ET, Qi X, Avella DM, Li N, Yang QX, Kester M, Rountree CB, Kaifi JT, Cole DJ, Rockey DC (2017). Successful chemoimmunotherapy against hepatocellular cancer in a novel murine model. J Hepatol.

[30] Bitzer M, Horger M, Giannini EG, Ganten TM, Wörns MA, Siveke JT, Dollinger MM, Gerken G, Scheulen ME, Wege H, Zagonel V, Cillo U, Trevisani F (2016). Resminostat plus sorafenib as second-line therapy of advanced hepatocellular carcinoma - The SHELTER study. J Hepatol.

[31] Goto K, Kato N (2017). Histone deacetylase inhibitor for the treatment of hepatocellular carcinoma: Chemoimmunotherapeutic perspective and prospects. J Hepatol.

[32] Chou TC, Talaly P (1977). A simple generalized equation for the analysis of multiple inhibitions of Michaelis-Menten kinetic systems. J Biol Chem.

[33] Goto K, Watashi K, Murata T, Hishiki T, Hijikata M, Shimotohno K (2006). Evaluation of the anti-hepatitis C virus effects of cyclophilin inhibitors, cyclosporin A, and NIM811. Biochem Biophys Res Commun.

[34] Goto K, Lin W, Zhang L, Jilg N, Shao RX, Schaefer EA, Zhao H, Fusco DN, Peng LF, Kato N, Chung RT (2013). The AMPK-related kinase SNARK regulates hepatitis C virus replication and pathogenesis through enhancement of TGF-beta signaling. J Hepatol.

