# Supplementary material for: Novel therapeutic features of disulfiram against hepatocellular carcinoma cells with inhibitory effects on a disintegrin and metalloproteinase 10
Source: Oncotarget. 2018 Apr 10;9(27):18821–31. doi: 10.18632/oncotarget.24568 (PMC5922358; doi:10.18632/oncotarget.24568)
Supplement: Supplementary file 1 [file oncotarget-09-18821-s001.pdf]

Novel therapeutic features of disulfiram against hepatocellular carcinoma cells with inhibitory effects on a disintegrin and metalloproteinase 10

SUPPLEMENTARY MATERIALS

Cells

Li7 cells (RIKEN BioResource Center, Tsukuba, Japan) and HLE cells (Japanese Collection of Research Bioresources Cell Bank, Osaka, Japan) were cultured according to the individual protocols at 37°C and 5 % CO_2.

RNAi

Control and ADAM10-targeted siRNAs were purchased from Dharmacon (Lafayette, CO). The siRNAs were transfected into cells using Lipofectamine RNAiMAX Reagent (Thermo Fisher Scientific, Waltham, MA), as described previously [4].

NK cell cytotoxicity assay

NK cell-mediated cytotoxicity toward target hepatoma cells was determined with an LDH cytotoxicity detection kit (Takara Bio, Shiga, Japan) according to the manufacturer's protocol as described previously [5]. In brief, NK92MI cells were primed with 50 ng/mL IL-15 (R&D Systems) for 24 h and PLC/PRF/5 cells were pretreated with 10 µM DSF for 48 h, followed by co-culture at the E:T ratio 20:1 for 4 h and measurement of LDH release in the supernatants. The NK cell cytotoxicity was calculated with the following formula: Cytotoxicity (%) = 100 × [(Effector: Target cell mix − Effector cell control) − Low control]/(High control − Low control). For the high control, 0.5% Triton X-100 (Sigma-Aldrich) was used.

SUPPLEMENTARY REFERENCES

1. Chiba T, Suzuki E, Yuki K, Zen Y, Oshima M, Miyagi S, Saraya A, Koide S, Motoyama T, Ogasawara S, Ooka Y, Tawada A, Nakatsura T, Hayashi T, Yamashita T, Kaneko S, et al. Disulfiram eradicates tumor-initiating hepatocellular carcinoma cells in ROS-p38 MAPK pathway-dependent and -independent manners. PLoS One. 2014;9:e84807.
2. Wang Z, Tan J, McConville C, Kannappan V, Tawari PE, Brown J, Ding J, Armesilla AL, Irache JM, Mei QB, Tan Y, Liu Y, Jiang W, Bian XW, Wang W. Poly lactic-co-glycolic acid controlled delivery of disulfiram to target liver cancer stem-like cells. Nanomedicine. 2017;13:641-657.
3. Li Y, Wang LH, Zhang HT, Wang YT, Liu S, Zhou WL, Yuan XZ, Li TY, Wu CF, Yang JY. Disulfiram combined with copper inhibits metastasis and epithelial-mesenchymal transition in hepatocellular carcinoma through the NF-kappaB and TGF-beta pathways. J Cell Mol Med. 2018;22:439-451.
4. Goto K, Kato N, Chung RT. Anti-hepatocellular carcinoma properties of the anti-alcoholism drug disulfiram discovered to enzymatically inhibit the AMPK-related kinase SNARK *in vitro*. Oncotarget. 2016; 7:74987-74999. https://doi.org/10.18632/oncotarget.11820.
5. Goto K, Annan DA, Morita T, Li W, Muroyama R, Matsubara Y, Ito S, Nakagawa R, Tanoue Y, Jinushi M, Kato N. Novel chemoimmunotherapeutic strategy for hepatocellular carcinoma based on a genome-wide association study. Sci Rep. 2016; 6:38407.

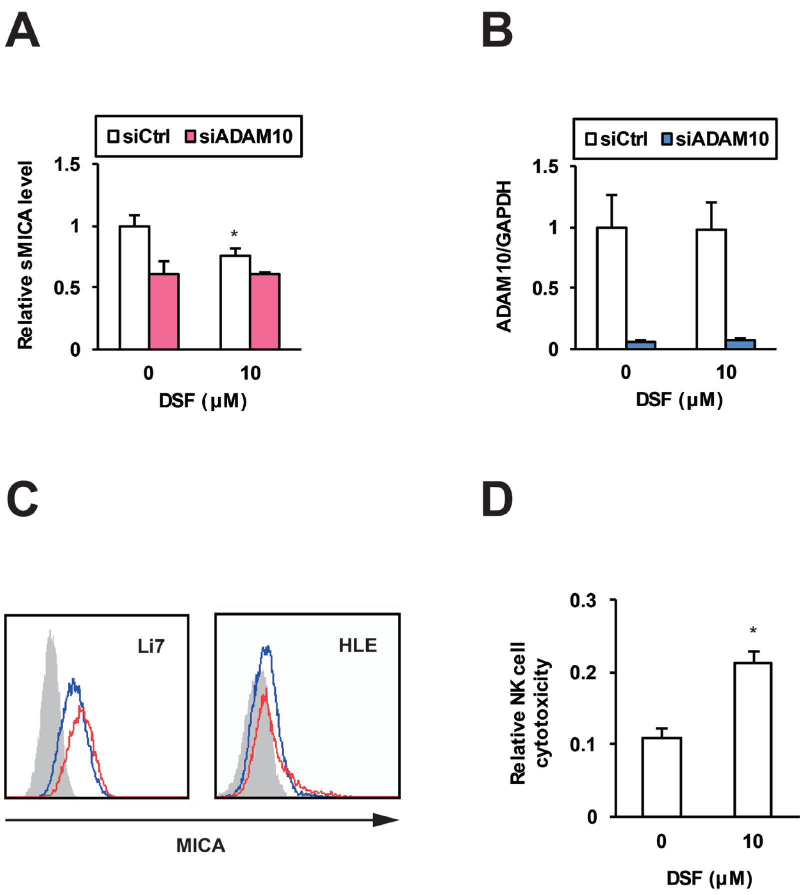

Supplementary Figure 1: ADAM10-targeted effects of DSF and NK cell cytotoxicity. After the transfection of PLC/PRF/5 cells with siRNA to ADAM10 (siADAM10) for 72 h, followed by the treatment with DSF at 10 μM for 48 h, sMICA levels were examined **(A)**, with the mRNA levels of ADAM10 determined by qRT-PCR normalized to *GAPDH* **(B)**. **(C)** After the treatment of Li7 and HLE cells for 72 h, the effects of DSF (0 and 15 μM in blue and red, respectively) on mMICA levels were evaluated flow cytometrically, with the isotype controls displayed as gray histograms. **(D)** PLC/PRF/5 cells pretreated with DSF at 10 μM for 48 h were cocultured with preprimed NK92MI cells at the effector:target ratio 20:1 for 4 h, followed by measurement of the level of LDH release in the culture medium. Relative NK cell activities were calculated as described in the Supplementary Materials and Methods. $^{*}P < 0.05$ by the Student's t-test.

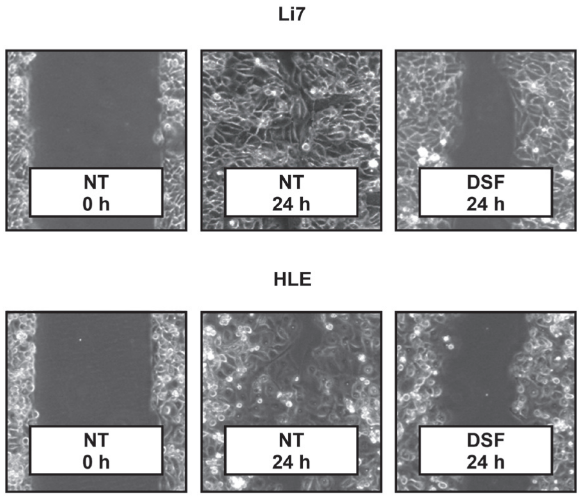

Supplementary Figure 2: Effects of DSF in multiple hepatoma cell lines. Li7 and HLE cells were pretreated with mitomycin at 10 μg/mL for 1 h, followed by the treatment with DSF at 15 μM for 24 h. NT, no treatment.

Supplementary Table 1: Relative mean fluorescence intensity

Compound	Dose (μM)	Relative intensity*
DSF	5	1.37
DSF	15	1.67
TR	5	1.48
TR	15	1.82
TMTM	15	0.94
DDC	15	1.01

* Relative to no treatment.

Supplementary Table 2: Anti-HCC effects of DSF *in vivo*

Effect	Mice	Hepatoma cells	Reference
Growth	NOD/SCID	Huh1, Huh7	Chiba *et al*. [1]
Growth/Metastasis	BALB/c-Nu/Nu	PLC/PRF/5	Wang *et al*. [2]
Metastasis	BALB/c	Hep3B	Li *et al*. [3]
